# Recent Advances in the Determination of Pesticides in Environmental Samples by Capillary Electrophoresis

**DOI:** 10.3390/ijerph13040409

**Published:** 2016-04-08

**Authors:** Po-Ling Chang, Ming-Mu Hsieh, Tai-Chia Chiu

**Affiliations:** 1 Department of Chemistry, Tunghai University, Taichung 40704, Taiwan; poling@thu.edu.tw; 2 Department of Chemistry, National Kaohsiung Normal University, 62, Shenjhong Road, Yanchao District, Kaohsiung 82446, Taiwan; 3 Department of Applied Science, National Taitung University, 369, Section 2, University Road, Taitung 95092, Taiwan

**Keywords:** capillary electrophoresis, environmental samples, pesticides, preconcentration, sample pretreatments

## Abstract

Nowadays, owing to the increasing population and the attempts to satisfy its needs, pesticides are widely applied to control the quantity and quality of agricultural products. However, the presence of pesticide residues and their metabolites in environmental samples is hazardous to the health of humans and all other living organisms. Thus, monitoring these compounds is extremely important to ensure that only permitted levels of pesticide are consumed. To this end, fast, reliable, and environmentally friendly methods that can accurately analyze dilute, complex samples containing both parent substances and their metabolites are required. Focusing primarily on research published since 2010, this review summarizes the use of various sample pretreatment techniques to extract pesticides from various matrices, combined with on-line preconcentration strategies for sensitivity improvement, and subsequent capillary electrophoresis analysis.

## 1. Introduction

Modern agricultural production depends heavily on the use of agrochemicals, including both synthetic and natural pesticides. A large number of pesticide classes and families are widely used to control insects, fungi, bacteria, weeds, nematodes, rodents, and other pests to maximize the harvest. These pesticides originally came into common use as a means of eliminating pests and limiting their adverse effects in agriculture, the household, and various other aspects of life [1,2]. Currently, over 800 active ingredients are used in nearly 3000 commercial products sold worldwide [3,4]. Because of the increasing population and its associated needs, the range of pesticide applications is continually expanding; with this rapid increase in their use, more and more pesticides are entering into the environment through various ways. These compounds are not only toxic, but also mobile and capable of bioaccumulation. Furthermore, pesticides also influence various physical, chemical, and biological processes. Exposure to contaminated environmental samples, including food, water, and soil may be harmful to the health of not only humans, but also of all other living organisms [5,6,7,8]. Thus, the concentration levels of pesticides and their metabolites in the environment must be continuously monitored. Maximal residue limits (MRLs) for pesticides have been established by the United Nation’s Food and Agriculture Organization and the World Health Organization [9]. In recent years, some MRL limits have been lowered from ppm to sub-ppm levels to satisfy the increasing demand for public health protection. Therefore, to protect humans from possible harm caused by pesticides, reliable methods that can analyze dilute mixtures of parent substances and their metabolites are required.

Pesticide residues and their metabolites have been analyzed in environmental samples using a variety of chromatographic methods such as gas chromatography (GC), high performance liquid chromatography (HPLC), and capillary electrophoresis (CE) [10,11,12,13,14,15,16,17,18,19,20]. Due to their high sensitivity and selectivity, GC and HPLC are the most frequently used methods for the detection of these compounds. However, because many pesticides exhibit thermal instability and low volatility, derivatization reactions, tedious pretreatment procedures, and a large amount of organic solvent are required for GC and HPLC. As a result, CE has become the preferred alternative method for the analyses of pesticides and their degradation products [19,20]. CE is widely used for pesticide determination due to its high separation efficiency, short separation time, low reagent consumption, and ease of operation; however, its concentration sensitivity is low when coupled with UV–Vis detectors. To overcome this limitation, CE can be used in combination with sample offline pretreatment or on-line preconcentration techniques with high enrichment factors. This review article aims to describe the current status of various types of sample pretreatment techniques used for the extraction and subsequent CE screening of pesticide residues and their metabolites in different environmental samples (e.g., water, soil, fruits, and food), with a special emphasis on the works published in the last five years. 

## 2. Sample Pretreatment Techniques

The determination of pesticide residues in real samples is often well beyond the capability of CE. In some cases, pesticides appear in food and environmental samples at trace levels well below the detection capability of CE alongside a variety of chemicals in complicated sample matrices. Thus, reliable CE methods coupled with sample pretreatment and on-line preconcentration techniques are necessary. A number of sample pretreatment systems that purify and/or concentrate pesticides prior to analysis have been developed and reviewed [21,22,23,24,25,26,27,28,29,30,31,32,33,34]. Most sample pretreatment methods carried out prior to CE analysis typically involve an extraction procedure that isolates and enhances the analytes of interest in the sample matrix. Liquid-liquid extraction (LLE) is the most common preconcentration/matrix isolation technique in analytical chemistry [32]; another popular approach is solid phase extraction (SPE), which has also been developed for pesticide analysis [21,22]. Due to increasing demand for simple, rapid, and accurate sample preparation procedures for pesticides, the techniques are being developed, separation principles based on liquid phase extraction and SPE techniques. The following sections provide an overview of the applications of these sample pretreatment techniques such as dispersive liquid-liquid microextraction (DLLME), SPE, solid-phase microextraction (SPME), matrix solid-phase dispersion (MSPD), and quick, easy, cheap, effective, rugged, and safe (QuEChERS).

### 2.1. Dispersive Liquid-Liquid Microextraction

DLLME is based on a ternary component solvent system wherein a mixture of extraction and disperser solvents is rapidly injected into an aqueous sample to form a cloudy solution [34]. Extraction equilibrium is quickly achieved because of the extensive surface contact between the droplets of the extraction solvent and the sample. The analytes are then collected in the extraction solvent. After centrifugation, the extraction solvent is usually sedimented at the bottom of a conical test tube and collected using a microsyringe. The extraction solvent is then evaporated to dryness at room temperature and redissolved in water for CE analysis. Zhang *et al.* [35] reported a method for the multiresidue analysis of carbamate pesticides in apples by using DLLME coupled sweeping-micellar electrokinetic chromatography (MEKC). Chloroform gave the highest extraction efficiency and was selected as the extraction solvent. The best extraction recoveries were achieved when acetone was used as disperser solvent. This approach has comparable limit of detection (LOD) and relative standard derivation with other reported extraction methods, but requires much shorter extraction time (1 min). A similar sweeping-MEKC method combined with DLLME was used to prepared samples of carbamate pesticides in juices (methanol as disperser solvent and chloroform as extraction solvent) [36], and neonicotinoid insecticides in cucumber (acetonitrile as disperser solvent and chloroform as extraction solvent) [37]. Soisungnoen *et al.* [38] have developed a rapid and sensitive method using DLLME (acetonitrile as disperser solvent and dichloromethane as extraction solvent) coupled with MEKC for the analysis of five organophosphorus pesticides. An efficient method based on DLLME-MEKC has been developed for determination of 2,4-dichlorophenoxybutyric acid, dicamba and 2,4-dichlorophenoxyacetic acid in environmental water samples [39]. A DLLME-nonaqueous capillary electrophoresis method has been proposed for the determination of imazalil, prochloraz, and thiabendazole fungicides in fruits and juice samples [40].

DLLME has showed its potential to be an efficient green methodology and offers advantages of speed, simplicity and low consumption of organic solvent when compared with the other extraction methods. High enrichment factors are achieved from an aqueous phase containing the analytes using a minimal amount of organic solvent. The combination with capillary electrophoretic approaches allows the selectivity and sensitivity to be further improved.

### 2.2. Solid Phase Extraction

SPE is a physical extraction process that was first introduced in the mid-1970s [21]. SPE offers several significant advantages over LLE, such as less consumption of organic solvent, shorter analysis time, no phase emulsion, higher method recovery, and more efficient removal of interfering compounds. Conventional SPE involves two essential steps: adsorption of the analytes onto the stationary phase and desorption from the solid material using small amounts of a favorable elution solvent. Generally, SPE procedures used to prepare samples for CE analysis use disposable cartridges, most of which are packed with silica-based phases. C_18_ cartridges have been used for SPE of organophosphorus pesticides in vegetables and fruits [41], phenazine-1-carboxylic acid in soil samples [42], sulfonylurea herbicides in water and grape samples [43], neonicotinoid insecticides in water samples [44], and carbamate insecticides in spiked river water and soil samples [45]. The combinations of SPE and CE approaches have shown high reliability, accuracy, and sensitivity to trace concentrations of pesticide residues.

Recently, various nanomaterials have been used as novel sorbents for sample pretreatment with SPE and SPME [46,47,48]. Among them, carbon-based nanomaterials such as carbon nanotubes, graphene oxide, and nanodiamonds were reported as excellent solid phases for sorption. Other nanomaterials such as noble-metal nanoparticles and metal-oxide nanoparticles have also been used for the same purposes. Magnetic solid phases have further advantages, because they eliminate the need for centrifugation and filtration steps; in addition, the solid phase can be easily separated from the sample solution with the help of an external magnetic field [48]. 

Multiwalled carbon nanotubes have been used as sorbents for the extraction of metsulfuron methyl and chlorsulfuron in water samples [49]. Chlorophenoxy acid herbicides in water samples were extracted through SPE combined with electromembrane extraction and graphene oxide was used as sorbents [50]. N-doped TiO_2_ nanotube cartridges showed better enrichment ability for SPE of paraquat and diquat in water samples [51]. A magnetic SPE technique using iron oxide nanoparticles immobilized with Ti^4+^ using polydopamine as bridge molecules was used to extract glyphosate and aminomethylphosphonic acid in river water [52]. This work reveals that the magnetic SPE based on immobilized metal affinity extraction is helpful for preparation of batches of field samples.

### 2.3. Solid-Phase Microextraction

SPME has been introduced as an alternative to traditional sample preparation techniques, because it provides a rapid, simple, effective, solvent-free, and sensitive pretreatment method and can also be easily combined with various separation techniques [53,54,55]. The basic SPME device consists of a fused silica fiber (or a metal core) coated with an appropriate stationary phase. The fiber is fixed inside a needle of the syringe-like device. Extraction is performed either by immersing the fiber in the gaseous or relatively pure liquid medium, or by sampling the analytes from the headspace above the investigated medium. Chen *et al.* [56] developed a rapid, selective, and efficient method for dispersive SPME using microbeads composed of a molecularly imprinted polymer for the determination of sulfamethzine in milk samples. The molecularly imprinted polymer microbeads showed a faster rate (reduced to 5 min) to achieve adsorption equilibration when compared with those of the non-imprinted polymer microbeads. However, the use of SPME has some drawbacks. Mainly, commercially available SPME fibers are expensive and have limited lifetime, since they tend to degrade with increased usage. The difference in length and thickness of SPME fiber coatings may result in variation of analyte enrichment from fiber to fiber. These disadvantages limited its further applications.

### 2.4. Matrix Solid-Phase Dispersion

MSPD was first introduced by Baker *et al.* [57], extraction and clean-up are integrated in a single step, thus making the procedure simple, fast, low-cost, less sample loss and solvent consumption. This technique involves mixing or blending (depending on the sample state) a sample with an appropriate sorbent such as Florisil, C18, alumina, or silica, until a homogeneous mixture is obtained. Wang *et al.* [58] used dichloromethane as extractant and C18-bonded silica as sorbent because its non-polar characteristic provided the best recovery (>90%) for three phenylurea herbicides in rice powders. The MSPD procedure was also developed to handle phenylurea herbicides in yam samples [59]. MSPD is more applicable to the pretreatment of solid samples rather than other sample pretreated techniques. However, the drawbacks of MSPD technique are the need for manipulation, the large number of variables (*i.e.*, sample amount, the amount and type of dispersant material, clean-up step, and composition of eluent) to be optimized.

### 2.5. Quick, Easy, Cheap, Effective, Rugged, and Safe

The QuEChERS procedure was developed by Anastassiades and coworkers in 2003 as a new approach to extract pesticides from fruits and vegetables [60]. This method was introduced as a green, user-friendly, and cheap approach to meet the changing needs of multiresidue analysis with good recovery and reproducibility. QuEChERS is based on a liquid partition with organic solvent, followed by a dispersive SPE for cleanup [15]. It has been adopted by many laboratories worldwide, and official methods are available from the Association of Official Analytical Chemists [61]. A CE-MS(mass spectrometry)/MS method for the determination of halosulfuron-methyl residue in sugarcane juice and tomato is introduced by Daniel *et al.* [62]. The samples were submitted to a QuEChERS extraction procedure followed by electrophoretic separation. A procedure combining QuEChERS with DLLME and sweeping-MEKC was developed for the determination of organophosphorus pesticides in *Astragalus membranaceus* [63]. The method was characterized with good resolution, great repeatability, and satisfactory recovery.

Sample preparation is a crucial step to the development of analytical methods for pesticides residue and their metabolites in complex environmental samples. Thus, miniaturization based on the traditional extraction methods is very important in order to reduce solvent volumes, wasted materials, time, and cost. These sample preparation techniques such as DLLME, SPE, MSPD, and QuEChERS provide good compatibility with CE for the determination of pesticide residue and their metabolites in complex samples. 

## 3. Pesticide Analysis Using Capillary Electrophoresis

CE represents one of the most attractive analytical techniques for the rapid qualitative and quantitative analysis of molecules with a wide range of polarities and molecular weights, including both small molecules such as amino acids and large macromolecules such as proteins and nucleic acids [64,65].

Because of its versatility and high separation efficiency, CE has become an interesting alternative to the widely used HPLC and GC and has gained considerable interest for pesticide analysis. Several excellent reviews on the various aspects of pesticide separation in different matrices using CE have been recently published [17,18,19,20]. Recent research on the use of CE to analyze pesticide residues and their metabolites in real samples based on different separation approaches and on-line preconcentration strategies are summarized in Table 1, Table 2, Table 3 and Table 4.

### 3.1. Capillary Zone Electrophoresis 

Capillary zone electrophoresis (CZE) is based on differences in the charge-to-mass ratios of the analytes during electrophoresis. CZE has been applied to the analysis of herbicides, among them glyphosate is a broad-spectrum, non-selective, post-emergence, and systemic organophosphorus herbicide that is used extensively in various applications for control of long grasses, weeds, and vegetation worldwide. The indiscriminate use of glyphosate increases the potential for its accumulation in the environment. Iwamuro *et al.* [66] demonstrated a CE-MS method using an amino group-modified capillary for the determination of glyphosate, glufosinate, bialaphos, aminomethylphosphonic acid, and 3-methylphosphinicopropionic acid in soil and tea beverages in 10 min. Cao *et al.* [68] developed a CE-laser-induced fluorescence (LIF) method using 5-(4,6-dichlorotriazinyl)amino fluorescein as the derviatization agent for the analysis of glyphosate, glufosinate, and aminomethylphosphonic acid in soil and water samples; the corresponding LODs were 3.21, 6.14, and 1.99 ng/kg, respectively.

Paraquat and diquat are typical quaternary ammonium herbicides. These herbicides are toxic to human beings and have been classified as moderately hazardous compounds by the World Health Organization [96]. Zhou *et al.* [73] reported a CZE method using an ionic liquid (1-butyl-3-methylimidazolium hexafluorophosphate) as an electrolyte for the determination of paraquat and diquat. Wuethrich *et al.* [74] reported a green sample preparation device based on the use of an electric field to transfer the analytes from a large volume of sample into small volumes of electrolyte suspended in two glass micropipettes using a conductive hydrogel. The device is designed to be used for the simultaneous electrophoretic sample concentration and separation of cationic and anionic analytes. In combination with CZE, this device was successfully applied to the analysis of herbicides (paraquat, diquat, and difenzoquat) in river and drinking water samples. The LODs for all paraquat, diquat, and difenzoquat were low (0.5 ng/mL).

### 3.2. Micellar Electrokinetic Chromatography

Micellar electrokinetic chromatography (MEKC) has become a powerful separation technique for both neutral and ionic compounds in complicated mixtures containing a broad range of analytes. MEKC is based on the differential partitioning between the micellar and aqueous phases. A method to determine glyphosate and aminomethylphosphonic acid in tap and river water though a dynamic supported liquid membrane tip extraction procedure followed by MEKC with capacitively coupled contactless conductivity detection (C^4^D) was first introduced by See *et al.* [69]. Low limits of detection (0.005 μg/L for glyphosate and 0.06 μg/L for aminomethylphosphonic acid) were achieved. Sung *et al.* [70] demonstrated a surface-sampling technique of in-line coupling liquid extraction surface analysis with CE to analyze the pesticides on solid surface. This direct surface analysis method was applied to determine organophosphorus pesticides on the external surface of apples. A rapid SPE-MEKC method of trifloxystrobin, tebufenozide, and halofenozide in foods has been developed [84]. The detection limits were between 0.088 and 0.094 mg/kg. Santalad *et al.* [45] reported a SPE-MEKC method for the determination of six carbamate insecticides. A 100-fold preconcentration factor was achieved with detection limits of 0.5 μM for methomyl and 0.01 μM for the other five pesticides. Chen *et al.* [83] has reported water-soluble CdTe/CdSe core-shell quantum dots were used for the selective fluorescence enhancement of organophosphorus pesticides. These compounds were subsequently analyzed by CE-LIF. The baseline separation was achieved in 12 min, and the obtained detection limits ranged from 50 to 180 μg/kg.

### 3.3. Other Approaches

Li *et al.* [81] demonstrated a method based on the combination of microemulsion electrokinetic chromatography and vortex-assisted surfactant-enhanced-emulsification liquid-liquid microextration for the determination of five triazine herbicides (simazine, atrazine, ametryn, prometryn, and terbutryn) in water samples. The detection limits of this method varied from 0.41 to 0.62 ng/mL. Microchip-CE has also reported for simple, disposable, rapid, and sensitive analysis of pesticide residues [71,72,77,78].

Phenylurea herbicides are widely used to selectively control weeds in a variety of crops. However, these compounds exhibit chronic toxicity, and their residues and degradation products are potential risks to human health through their accumulation in the environment and the food chain. Wang *et al.* [58] developed a new method based on MSPD-CE with enhanced chemiluminescence detection for the simultaneous determination of three phenylurea herbicides (isoproturon, linuron, and diuron). Poly-β-cyclodextrin was used to improve the separation resolution of the three analytes. These analytes were well separated within 8 min with LODs of 0.1 μg/L for isoproturon and linuron, and 0.2 μg/L for diuron. A similar CE-enhanced chemiluminescence system has been applied to detect three phenylurea herbicides (monuron, monolinuron, and diuron) in yam samples [59]. 

Tabani *et al.* [75] has reported that the low voltage electromembrane extraction combined with cyclodextrin modified CE was applied for the determination of 2,4-dichlorophenoxybutyric acid, 3,6-dichloro-2-methoxybenzoic acid, and 2,4-dichlorophenoxyacetic acid in river water. A CE method compatible with MS detection for simultaneously analyzing neonicotinoid insecticides in beeswax was reported by Sánchez-Hernández *et al.* [80]. Low LOD and limit of quantitation were achieved for all analytes. A CE immunoassay method with LIF detector was developed for the determination of metolcarb in rice and cucumber [81] and norfloxacin in food samples [85] with satisfactory recovery.

As shown in Table 1, Table 2 and Table 3, CZE and MEKC are the most successful CE approaches for the analysis of pesticide residues and their metabolites. Miniaturization based on the DLLME and SPE methods is of high importance in order to reduce solvent volumes, wasted material, time and cost. Recent sample preparation techniques such as SPE, DLLME, and QuEChERS coupled CE, have been demonstrated to be suitable for the determination of pesticide residues in complex environmental matrices.

## 4. On-Line Preconcentration of Pesticides by Capillary Electrophoresis

CE has advantages such as short analysis time, high separation efficiency, low reagent consumption, and low operation cost over other chromatographic methods. However, its poor sensitivity due to the short optical path length of CE with UV detection and the low injection volumes of the sample solution significantly limit the application of CE to the analyses of pesticide residues. Therefore, on-line focusing is the simplest way to achieve sample enrichment. To date, various on-line focusing strategies have been designed, such as large-volume sample stacking (LVSS), field-amplified sample injection (FASI), transient isotachophoresis, dynamic pH junction, and sweeping; all of these methods have been thoroughly reviewed [97,98,99,100,101]. As shown in Table 4, these on-line focusing strategies have been used for the determination of pesticides in different environmental samples. 

### 4.1. pH-Mediated Stacking

Dynamic pH junction is based on the creation of a pH discontinuity that is established by injecting the sample at a different pH than the BGE and can be used to concentrate weakly ionic analytes. Arribas *et al.* [86] demonstrated an analysis method for amitrol and triazine herbicides (atrazine, ametryn, and atraton) and a degradation product (2-hydroxyatrazine) in untreated water samples (mineral, spring, tap, and river water) by combining CZE-UV and acid-assisted on-column preconcentration. The resultant LODs were in the range of 54 to 310 nM. A normal moving reaction boundary-based stacking of phenazine-1-carboxylic acid followed by CZE was reported by Sun *et al.* [87]; the obtained LOD of phenazine-1-carboxylic acid in soil was decreased to 17 ng/g.

### 4.2. Field-Amplified Sample Stacking and Large-Volume Sample Stacking

Field-amplified sample stacking (FASS) is performed by hydrodynamically injecting a low-conductivity sample solution into the capillary filled with a high-conductivity separation solution. When the voltage is applied, sample ions migrate rapidly because the electric field is higher in the sample zone than in the background electrolytes. The stacking occurs at the sample and background electrolytes boundary and the ions are concentrated into a narrow zone. The difference between FASS and large-volume sample stacking (LVSS) is the injection volumes, LVSS sometimes can inject a sample solution up to the entire capillary volume. See *et al.* [89] compared two on-line preconcentration strategies (LVSS and field-enhanced sample injection) for the analysis of glyphosate, glufosinate, and aminophosphonic acid in drinking water by CE with C^4^D. By performing a field-enhanced sample injection-CE-C^4^D procedure, excellent LODs of 0.0005–0.02 μM were obtained along with 245–1002-fold sensitivity enhancements. A combination of ultrasonic extraction, Soxhlet extraction, and FASS-CE was developed for the sensitive determination of pyoluteorin in soil samples, and the obtained LOD was 0.107 μg/mL [90]. A sensitive method for the determination of trace residues of sulfonylurea herbicides in water and grape samples by LVSS-CZE was developed by Quesada-Molina *et al.* [43]. The obtained LODs for the studied compounds ranged from 0.04 to 0.12 μg/L for water samples and from 0.97 to 8.30 μg/kg for grape samples. Yi *et al.* [91] demonstrated a LVSS-MEKC method, with polarity switching, for the detection of sulfonylurea herbicides in cereals. Under optimum conditions, the LODs were in the range of 0.22 to 0.89 ng/g, and the sensitivity enrichment factors ranged from 570 to 835.

### 4.3. Sweeping

Sweeping involves interactions between a pseudostationary phase or a complexing agent in the separation buffer and the analytes in a matrix that does not contain additives. Accumulation of the analyte is caused by chromatographic partitioning, complexation, or any interaction between the analytes and additives during electrophoresis. Wang’s group combined DLLME and sweeping-MEKC for the analysis of pesticides such as carbamate in apples [35], sulfonylurea herbicides in soil samples [93], and neonicotinoid insecticides in cucumber samples [37]. A similar method developed by Moreno-González *et al.* [36] was used to determine carbamates in juice samples, and LODs ranging from 1 to 7 μg/L were obtained. Fang *et al.* [92] reported an on-line sweeping-MEKC method for the determination of the residues of eight triazine herbicides in cereal and vegetable samples. The obtained enrichment factors ranged from 479 to 610, and the LODs ranged from 0.02 to 0.04 ng/g. Santalad *et al.* [94] proposed a reversed-electrode polarity mode coupled MEKC for the analysis of carbamate insecticides residues in fruit juices. This method gave enrichment factors of about 4 to 13 and LODs of 0.01 to 0.10 mg/L.

### 4.4. Other Stacking Strategies

**Figure 1:**
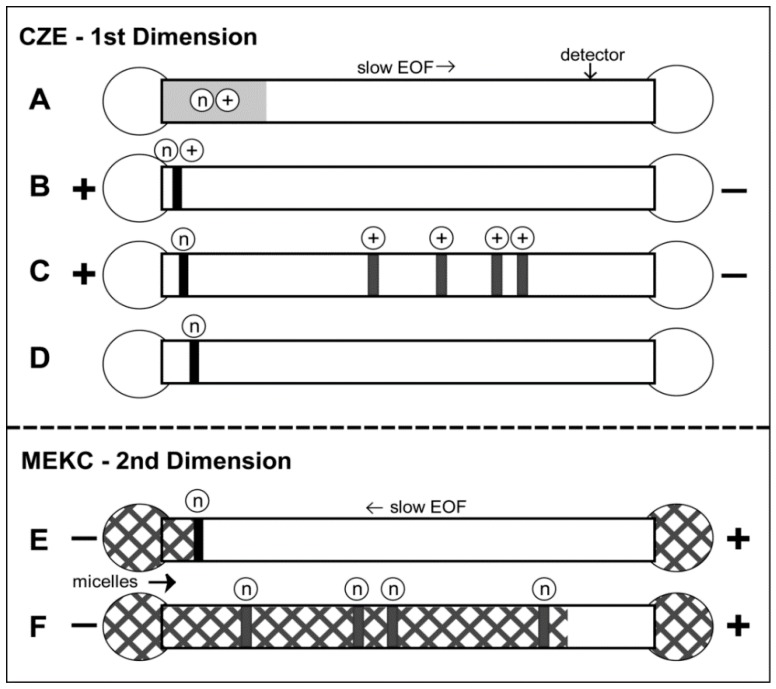
Stacking and separations of two different classes of compounds [cationic (+) and neutral (n) analytes] in interface-free 2-D heart-cutting CE (CZE × MEKC). (**A**) The fused-silica capillary was filled with a low-pH CZE electrolyte. The sample was injected as a long plug. The CZE electrolye was placed at both ends of the capillary; (**B**) A voltage was applied at positive polarity (cathode at the detector end). The analytes were focused by stacking; (**C**) Continued application of voltage caused the migration of the concentrated cationic analytes to the detector; (**D**) The cationic analytes migrated out of the capillary, and the concentrated neutral analytes were purified and remained inside the capillary. The first dimension analysis ended; (**E**) The start of the second dimension analysis was the replacement of the CZE electrolye at both ends of the capillary by the low-pH MEKC electrolyte with SDS micelles. The presence of micelles is depicted by the square patterned zones. Application of voltage at negative polarity (anode at detector end) caused the electrophoretic migration of the SDS micelles into the capillary. The micelles eventually penetrated or swept the neutral analyte fraction; (**F**) Continued application of voltage caused the micelle-bound analytes to separate and migrate to the detector. The second dimension and analysis ends when all the analytes were detected. Reprinted with permission from [95]. Copyright (2016) American Chemical Society.

Kulusamude *et al.* [95] employed interface-free, two-dimensional, heart-cutting CE for the analysis of cationic and neutral small molecules. The scheme is depicted in Figure 1. CZE and MEKC were employed in the first and second stages, respectively. In the first dimension of CZE, neutrals are not concentrated. Neutrals can be concentrated in the second dimension of MEKC *via* sweeping. The stacking mechanisms of sweeping and analyte focusing by micelle collapse were successfully used to analyze eight cationic drugs, five neutral steroids, three quaternary ammonium pesticides, and three neutral organophosphate pesticides with sensitivity enhancement factors ranging from 15 to 100.

Different stacking strategies have been used successfully for the pesticide determination in complex samples such as water, soil, fruits, vegetables, and foods. Also, the combination of on-line preconcentration approaches can provide greater enhancement factors than when a single method is used. A remarkable trend is also seen in combination with off-line pretreatment steps such as LLE, DLLME, SPE, and QuEChERS. Thus, the combination of off-line and on-line preconcentration techniques further improves the sensitivity in CE. The higher sensitivity the use of these approaches provides is of great importance in metabolomics studies because the trace concentration of metabolites in the environmental samples at short times after pesticides application.

## 5. Conclusions

The increasingly widespread application of pesticides means that ever larger amounts of them may be entering the environment and threatening human health. During their application, pesticides can accumulate in the environment, in surface and ground water, soil, food, and food products. They are usually present in very trace amounts and in matrices of great complexity. Thus, sample pretreatment and on-line concentration methods are needed to determine trace pesticide residues and their degradation products in the environment. Herein, we reviewed the most relevant CE applications for the determination of pesticides in various environmental samples such as vegetables, fruits, foods, soils and water, and the different sample pretreatment methods for pesticides in these matrices are also included.

Sample preparation is recognized as the most critical step in pesticide analysis if good accuracy, selectivity, sensitivity, and robustness are to be achieved. Over the past few decades significant efforts have been devoted to reducing time, cost, manual handling, and consumption of solvents and samples. Numerous innovative developments in respect of the liquid phase or miniaturized solid devices have been proposed. The combination of miniaturized sample preparation with CE has powerful potential in pesticide analysis.

Although remarkable achievements have been made in maximizing the detection sensitivity, substantial developmental challenges and opportunities still exist such as green and fast sample pretreatment with automation, high throughput and fast screening, and portable devices. For example, such a method would utilize a green sample preparation approach applicable to more complex matricies, and a portable equipment (microchip-CE or bio/nanosensors) [71,72,77,78,102,103,104] for the analysis, providing a real-time response of the pesticide residues. Due to the excellent selectivity and specificity of MS detection and the information provided about the molecular weights and structures of the solutes, more applications of CE-MS to the quantitative analysis of pesticides and their metabolites. This technique could help to establish their degradation pathways and evaluate the toxicity of the degradation products to establish the allowed MRLs. It should also be mentioned that the use of a suitable sample preparation procedure along with a suitable on-line preconcentration strategy for CE has allowed the achievement of LODs very similar to those obtained by chromatographic approaches. Due to the versatility, high efficiency and selectivity, CE can become a powerful alternative for fast pesticide screening.

## Figures and Tables

**Table 1:** Recent applications of capillary electrophoresis for the analysis of herbicides.

Analytes	Matrix	Pretreatment	CE mode	Detection	Separation Buffer	LOD	Ref.
Glyphosate, aminomethylphosphonic acid	River water, Round^®^	Fe_3_O_4_@PDA-Ti^4+^ nanoparticles based mSPE	CZE	DAD (203 nm)	25 mM tetraborate (pH 9.3)	0.4 ng/mL	[52]
Glyphosate, glufosinate, bialaphos, aminomethylphosphonic acid, 3-methylphosphinicopropionic acid	Soil, tea beverage	Filtration	CZE	MS	100 mM formic acid adjusted with 100 mM ammonia (pH 3.4)	0.5–10 μg/mL	[66]
Glyphosate, aminomethylphosphonic acid, glyoxylate, sarcosine, formaldehyde	*Lolium* spp.	LLE	CZE	DAD (indirect, 220 nm)	10mM potassium phthalate (pH 7.5), 0.5 mM CTAB, 10% ACN	0.1–0.2 μg/mL	[67]
Glyphosate, glufosinate, aminomethylphosphonic acid	Lake and tap water, soil	Filtration	CZE	LIF	30 mM boric acid (pH 9.5), 15 mM Brij-35	1.99–6.14 ng/kg	[68]
Glyphosate, aminomethylphosphonic acid	Tap and river water	SLMTE	MEKC	C^4^D	12mM histidine, 8 mM MES (pH 6.3), 75 μM CTAB, 3% methanol	0.06–0.005 μg/L	[69]
Glyphosate, glufosinate-ammonium, aminomethylphosphonic acid	Apple surface	LE	MEKC	LIF (520 nm)	10 mM sodium tetraborate (pH 9.90), 10 mM SDS,10% (v/v) ACN	1–10 ppb	[70]
Glyphosate	Tap water	Online ITP	microchip CE	C^4^D	10 mM MES, Bis-Tris (pH 6.1), 0.1% MHEC	2.5 μg/L	[71]
Glyphosate, glufosinate	River water, broccoli, soybean	Water: filtration; broccoli, soybean: LLE	microchip CE	LIF	10 mM tetraborate (pH 9.0), 2% (w/v) HPC	0.02–0.05 μg/L	[72]
Paraquat, diquat			CZE	DAD (220 nm, 254 nm)	50 mM 1-butyl-3-methylimidazolium hexafluorophophate (pH 5.0), 10% ethanol	N.D.	[73]
Paraquat, diquat, difenzoquat	Tap and river water	SECS	CZE	UV (200 nm)	150 mM phosphate (pH 2.4)	0.5 ng/mL	[74]
Paraquat, diquat	Tap and mountain water	N doped TiO_2_ nanotube based SPE	CZE	DAD (220 nm, 254 nm)	50 mM 1-butyl-3-methylimidazolium hexafluorophophate, 10% ethanol (pH 5.0)	1.95–2.59 μg/L	[51]
Isoproturon, linuron, diuron	Vegetables, rice	MSPD	CE	ECL	20 mM phosphate (pH 7.5), 12 mg/mL poly-β-CD	0.1–0.2 μg/L	[58]
Monuron, monolinuron, diuron	*yam*	MSPD	CZE	ECL	25 mM phosphate (pH 8.0)	0.01–0.05 μg/L	[59]
Halosulfuron-methyl	Sugarcane juice, tomato	QuEChERS	CZE	MS	20 mM NH_4_HCO_3_ (pH 8.5)	2 ppb	[62]
Metsulfuron methyl, chlorsulfuron	Lake, creek, reservoir, underground water	MWCNT based SPE	CZE	DAD (231 nm)	50 mM tetraborate (pH 9.0), 3% methanol	0.36–0.40 μg/L	[49]
2,4-dichlorophenoxybutyric acid, 3,6-dichloro-2-methoxybenzoic acid, 2,4-dichlorophenoxyacetic acid	River water	Low-voltage-EME	CE	UV (214 nm)	100 mM phosphate (pH 9.0), 1 mM α-CD	10–15 ng/mL	[75]
2-methyl-4-chlorophenoxyacetic acid, 2-(2,4-dichlorophenoxy) propanoic acid, 2-(4-chloro-2-methylphenoxy) propanoicacid	River and sea water	Graphene oxide based SPE-EME	CZE	UV (214 nm)	75 mM phosphate (pH 9.0)	0.3–0.5 ng/mL	[50]
2,4-dichlorophenoxybutyric acid, 3,6-dichloro-2-methoxybenzoic acid, 2,4-dichlorophenoxyacetic acid	Lake, river, reservoir water	DLLME	MEKC	DAD (230 nm)	10 mM tetraborate (pH 9.75), 25 mM SDS, 15% (v/v) methanol	1.56–1.91 ng/mL	[39]
Atrazine, simazine, ametryn prometryn, terbutryn	Well, river, reservoir water	VSLLME	MEEKC	UV (220 nm)	10 mM borate (pH 9.5), 2.5% (w/v) SDS, 0.8% (w/v) ethyl acetate, 6.0% (w/v) 1-butanol	0.41–0.62 ng/mL	[76]
Atrazine, simazine, ametryn	Soil		microchip CE	Amperometry (pulsed)	1.5% agarose, 200 mM KCl in methanol:H_2_O (1:1)	0.36–0.55 nM	[77]
Atrazine, simazine, ametryn	Soil	LLE	microchip CE	Amperometry	1.5% agarose, 200 mM KCl in methanol:H_2_O (1:1)	0.36–0.55 nM	[78]

ACN: acetonitrile; C^4^D: capacitively coupled contactless conductivity detection; CD: cyclodextrin; CE: capillary electrophoresis; CTAB: cetyltrimethylammonium bromide; CZE: capillary zone electrophoresis; DAD: diode array detector; DLLME: dispersive liquid-liquid microextraction; ECL: enhanced chemiluminescence; EME: electro membrane extraction; HPC: hydroxypropyl cellulose; ITP: isotachophoresis; LIF: laser-induced fluorescence; LLE: liquid-liquid extraction; LOD: limit of detection; MEEKC: microemulsion electrokinetic chromatography; MEKC: micellar electrokinetic chromatography; MES: 2-(*N*-morpholino)ethanesulfonic acid; MHEC: methylhydroxyethylcellulose; MS: mass spectrometry; MSPD: matrix solid-phase dispersion; mSPE: magnetic solid phase extraction; MWCNT: multiwalled carbon nanotubes; N.D.: not determined; PDA: polydopamine; Ref.: reference; SDS: sodium dodecyl sulfate; SECS: simultaneous electrophoretic sample concentration and separation; SLMTE: supported liquid membrane tip extraction; SPE: solid phase extraction; VSLLME: vortex-assisted surfactant-enhanced-emulsification liquid–liquid microextraction.

**Table 2:** Recent applications of capillary electrophoresis for the analysis of insecticides.

Analytes	Matrix	Pretreatment	CE Mode	Detection	Separation Buffer	LOD	Ref.
Cyromazine	Pig and chicken feed, milk, egg	LLE	CZE	DAD (214 nm)	50 mM phosphate (pH 3.1)	0.12–0.13 mg/kg	[79]
Acetamiprid, clothianidin, dinotefuran, imidacloprid, nitenpyram, thiacloprid, thiamethoxam	Beeswax	LLE	CE	MS	0.5 M ammonia	1.0–2.3 μg/L	[80]
Acetamiprid, thiamethoxan, imidacloprid, 6-chloronicotinic acid	Drinking and river water, soil	Water: SPE Soil: MSPD	MEKC	DAD (254 nm)	5 mM borate (pH 10.4), 40 mM SDS, 5%(v/v) methanol	0.103–0.810 mg/L	[44]
Metolcarb	Rice, cucumber	Filtration	CE	LIF (520 nm)	20 mM Na_2_B_4_O_7_/10 mM NaH_2_PO_4_ (pH 9.0)	0.07 μg/L	[81]
Methomyl, carbaryl, carbofuran, propoxur, isoprocarb, promecarb	River water, soil	SPE	MEKC	Amperometry	20 mM tetraborate (pH 10.2), 20 mM SDS	0.1–3 μM	[45]
Carbofuran, carbosulfan, isoprocarb, 3-hydroxycarbofuran, 3-ketocarbofuran	Rice	LLE	MEKC	UV (200 nm)	20 mM phosphate (pH 8.0), 15 mM SDS	0.3–4.0 μM	[82]
Mevinphos, phosalone, methidathion, diazinon	Tomato	LLE	MEKC	LIF (532 nm)	30 mM tetraborate (pH 9.6), 50 mM SDS, 3% methanol	50–180 μg/kg	[83]
Methyl parathion, ethyl parathion, chlorpyrifos, chlorpyrifos-methyl, dimethoate, trichlorfon	Cabbage, white radish, grape, pear, orange	LLE-SPE	CEC	Amperometry (indirect)	0.1 mM DHBA, 50% (v/v) ACN 50% (v/v) 10 mM MES (pH 5.5)	0.008–0.2 mg/kg	[41]

ACN: acetonitrile; CE: capillary electrophoresis; CEC: capillary electrochromatography; CTAB: cetyltrimethylammonium bromide; CZE: capillary zone electrophoresis; DAD: diode array detector; DHBA: 3,4-dihydroxybenzylamine; LIF: laser-induced fluorescence; LLE: liquid-liquid extraction; LOD: limit of detection; MEKC: micellar electrokinetic chromatography; MS: mass spectrometry; MSPD: matrix solid-phase dispersion; Ref.: reference; SDS: sodium dodecyl sulfate; SPE: solid phase extraction.

**Table 3:** Recent applications of capillary electrophoresis for the analysis of fungicides.

Analytes	Matrix	Pretreatment	CE Mode	Detection	Separation Buffer	LOD	Ref.
Trifloxystrobin, tubefenozide, halofenozide	Tomato, celery, apple juices	SPE	MEKC	UV (202 nm)	10 mM tetraborate (pH 9.0), 18 mM SDS, 22.5% (v/v) ACN	0.088–0.094 mg/kg	[84]
Imazalil, prochloraz, thiabendazole	Apple, cherry tomato, grape juice	DLLME	NACE	UV (204 nm)	Methanol-ACN mixture (35:65 v/v) containing 30 mM NH_4_Cl, 0.5% (v/v) H_3_PO_4_	0.47–0.72 μg/kg	[40]
Sulfamethazine	Milk	MIP-DSPME	CZE	UV (267 nm)	10 mM tetraborate (pH 9.1)	1.1 μg/L	[56]
Norfloxacin	Chicken, pork, fish, milk	LLE	CE	LIF (520 nm)	30 mM Na_2_B_4_O_7_/NaH_2_PO_4_ (pH 9.0)	0.005 μg/L	[85]

ACN: acetonitrile; CE: capillary electrophoresis; CZE: capillary zone electrophoresis; DLLME: dispersive liquid-liquid microextraction; LIF: laser-induced fluorescence; LLE: liquid-liquid extraction; LOD: limit of detection; MEKC: micellar electrokinetic chromatography; MIP-DSPME: molecular imprinted dispersive sloid-phase microextraction; NACE: nonaqueous capillary electrophoresis; Ref.: reference; SDS: sodium dodecyl sulfate; SPE: solid phase extraction.

**Table 4:** Recent applications of on-line preconcentration of pesticides by capillary electrophoresis.

Analytes	Matrix	Pretreatment	CE Method	Detection	LOD	EF	Ref.
Amitrol, atrazine, ametryn, atraton, 2-hydroxyatrazine	Mineral, spring, tap, river water	Filtration	pH-mediated-CZE	UV (200 nm), amperometry	UV: 0.054–0.31 μM amperometry: 9.6 nM (amitrol)	N.D.	[86]
Phenazine-1-carboxylic acid	Soil	LLE	MRB-CE	UV (248 nm)	17 ng/g	214	[87]
(4-chloro-2-methylphenoxy)acetic acid, (*R*)-2-(2,4-dichlorophenoxy)- propanoic acid		Untreated	MSS-CZE	UV (210, 214, 240 nm)	0.06–0.12 μg/L	59–110	[88]
Glyphosate, glufosinate, aminophosphonic acid	Tap water	Untreated	LVSS-CE FESI-CE	C^4^D	0.1–2.2 μg/L	245–1002	[89]
Phenazine-1-carboxylic acid	Soil	SPE	FASS-CZE	UV (254 nm)	0.021 μg/L	N.D.	[42]
Pylouteorin	Soil	LLE-Soxhlet extraction	FASS-CE	UV (214 nm)	0.107 μg/mL	N.D.	[90]
Triasulfuron, rimsulfuron, flazasulfuron, metsulfuron-methyl, chlorsulfuron	Ground water, grape	SPE	LVSS-CZE	DAD (226 nm, 240 nm)	water: 0.04–0.12 μg/L grape: 0.97–8.30 μg/kg	N.D.	[43]
Nicosulfuron, thifensulfuon methyl , tribenuron methyl, sulfometuron methyl, pyrazosulfuron ethyl, chlorimuron ethyl	Rice, flour oatmeal, wholemeal	LLE	LVSS-MEKC with polarity switching	UV (254 nm)	0.22–0.89 ng/g	570– 835	[91]
Simazine, atrazine, simetryn, propazine, ametryn, terbuthylazine, prometryn, terbutryn	Cereal, chives, carrots	LLE	Sweeping-MEKC	DAD (220 nm)	0.02–0.04 ng/g	479–610	[92]
Methiocarb, fenobcarb, diethofencarb, carbaryl, isoprocarb, tsumacide	Apples	DLLME	Sweeping-MEKC	DAD (200 nm)	2.0–3.0 ng/g	491–1834	[35]
Carbofuran, carbaryl, methiocarb, promecarb, oxamyl, aldicarb, methomyl, baygon, asulam, benomyl, napropamid, carbendazim	Banana juice, pineapple juice, tomato juice	DLLME	Sweeping-MEKC	DAD (210 nm)	1–7 μg/L	N.D.	[36]
Chlorimuron ethyl, bensulfuron methyl, tribenuron methyl, chlorsulfuron, metsulfuron methyl	Soil	DSPE-DLLME	Sweeping-MEKC	DAD (220 nm)	0.5–1. 0 ng/g	3000–5000	[93]
Thiacloprid, acetamiprid, imidaclothiz, imidacloprid	Cucumber	DLLME	Sweeping-MEKC	DAD (243 nm, 268 nm)	0.8–1.20 ng/g	4000–10,000	[37]
Dimethoate, phosphamidon, paraoxon-methyl, paraoxon, fensulfothion	*Astragalus membranaceus*	QuEChERS-DLLME	Sweeping-MEKC	DAD (200 nm)	0.010–0.018 μg/mL	90.0–167.3	[63]
Parathion-methyl, malathion, diazinon, azin- phos-methyl, fenitrothion	Tap, surface water	DLLME	REPSM-MEKC	DAD (200 nm)	3–15 ng/mL	477–635	[38]
Methomyl, propoxur, carbofuran, carbaryl, isoprocarb, promecarb	Mangosteen, pomegranate, orange, apple, guava, kiwi, passion fruit juices	Filtration	REPSM-MEKC	DAD (205, 214, 225 nm)	0.01–0.10 mg/L	4.2–12.3	[94]
Diquat, paraquat, difenzoquat, parathion, fenitrothion, azinphos-methyl	Water	Untreated	Sweeping with AFMC-interface-free 2-D heart-cutting-CE	UV (200 nm)	0.004–0.02 μg/mL	15–100	[95]

AFMC: analyte focusing by micelle collapse; C^4^D: capacitively coupled contactless conductivity detection; CE: capillary electrophoresis; CZE: capillary zone electrophoresis; DAD: diode array detector; DLLME: dispersive liquid-liquid microextraction; DSPE: dispersive solid-phase extraction; EF: enhancement factor; FASS: field amplified sample stacking; FESI: field-enhanced sample injection; LLE: liquid-liquid extraction; LOD: limit of detection; LVSS: large volume sample stacking; MEKC: micellar electrokinetic chromatography; MRB: moving reaction boundary; MSS: micelle to solvent stacking, N.D.: not determined; QuEChERS: quick, easy, cheap, effective, rugged, and safe; Ref.: reference; REPSM: reversed electrode polarity stacking mode; SPE: solid phase extraction.

## Abbreviations

The following abbreviations are used in this manuscript:

C^4^D	Capacitively coupled contactless conductivity detection
CE	Capillary electrophoresis
CZE	Capillary zone electrophoresis
DLLME	Dispersive liquid-liquid microextraction
FASS	Field-amplified sample stacking
GC	Gas chromatography
HPLC	High performance liquid chromatography
LIF	Laser-induced fluorescence
LLE	Liquid-liquid extraction
LOD	Limit of detection
LVSS	Large-volume sample stacking
MEKC	Micellar electrokinetic chromatography
MRLs	Maximal residue limits
MS	Mass spectrometry
MSPD	Matrix solid-phase dispersion
QuEChERS	Quick, easy, cheap, effective, rugged, and safe
SDS	Sodium dodecyl sulfate
SPE	Solid phase extraction
SPME	Solid-phase microextraction
